# Sex differences in the effects of adolescent stress in an Alzheimer’s disease mouse model

**DOI:** 10.1002/alz.089305

**Published:** 2025-01-03

**Authors:** Emily Groom, Christina A Thrasher, Ava Herzog, Damian G Zuloaga, Kristen L Zuloaga

**Affiliations:** ^1^ Albany Medical College, Albany, NY USA; ^2^ State University of New York at Albany, Albany, NY USA

## Abstract

**Background:**

Stress is a common modifiable risk factor for AD, which increases dementia risk 2‐fold. During the stress response, the hypothalamic‐pituitary adrenal (HPA) axis is activated which stimulates the release of stress hormones called glucocorticoids into the blood stream. Studies on early‐life stress have shown a glucocorticoid dependent vulnerability towards late‐life inflammation. However, adolescence may be a more critical period for stress‐induced vulnerability for late‐life neurodegenerative diseases given that adolescents experience longer circulating levels of glucocorticoids following stress exposure and greater stress responsivity. Additionally, sex is an important biological variable because there are sex differences in AD prevalence and stress vulnerability with women being affected more than men. We hypothesized that adolescent stress would exacerbate AD pathology later in life and that this effect will be stronger in females.

**Method:**

Male and female WT and Tg‐SwDI mice underwent a chronic social stress paradigm during adolescence (1‐1.5 months of age). The paradigm consists of alternating social isolation and change in group composition for 20 days. Mice were assessed longitudinally at 2, 4, and 6 months of age for anxiety‐like behavior, cognitive function, and social behavior. Brains were collected at 6 months of age to assess neuropathology.

**Result:**

Adolescent stress had an age‐dependent effect on anxiety‐like behavior and cognitive function whereby mice who experienced adolescent stress had greater anxiety and worse cognitive function as they aged compared to control mice. Further, there were sex differences in both anxiety‐like behavior and social behavior at 6 months of age, with females showing greater anxiety‐like behavior and social avoidance compared to male mice. Neuropathology assessments are ongoing.

**Conclusion:**

This data suggests that adolescent stress may exacerbate AD pathology later in life, with females being more susceptible than males to these effects. This data is in line with clinical studies showing that females are more likely to develop AD and suffer from anxiety disorders.

